# Pharmacokinetics and bioequivalence assessment of optimized directly compressible Aceclofenac (100 mg) tablet formulation in healthy human subjects

**DOI:** 10.1371/journal.pone.0238951

**Published:** 2020-09-08

**Authors:** Rabia Bushra, Muhammad Harris Shoaib, Huma Ali, Sana Ghayas

**Affiliations:** 1 Department of Pharmaceutics, Faculty of Pharmacy and Pharmaceutical Sciences, University of Karachi, Karachi, Pakistan; 2 Department of Pharmaceutics and Bioavailability and Bioequivalence Research Facility, Faculty of Pharmacy and Pharmaceutical Sciences, University of Karachi, Karachi, Pakistan; Bhagwan Mahvir College of Pharmacy, INDIA

## Abstract

The aim of the study was to determine the various pharmacokinetic parameters of the newly developed cost-effective aceclofenac 100 mg tablet formulation (F-15) and to establish the bioequivalence against the marketed brand (ACEMED). Both products (test and reference) were given to 12 healthy non-smokers male subjects with overnight fasting of >10hr. The study was a randomized, single-dose, open-label, two sequence, and two treatment crossover design, with a washout period of 2 weeks. Blood samples (5 mL) from the human subjects were collected before (0 hr) and after drug administration at 13different time points (0.5, 1, 1.5, 2, 2.5, 3, 3.5, 4, 5, 6, 8, 12 and 18 hrs). The drug plasma concentration was analyzed by a validated RP-HPLC method using a solvent system containing acetonitrile and deionized water (60:40% v/v). Linearity was found to be 0.999 over the drug concentration range of 50μg/mL to 0.05μg/mL with LLOQ and LOD of 0.05μg/mL and 0.025μg/mL respectively. Non-compartmental pharmacokinetic analysis was performed using Kinetica^®^ (ver. 5.1) software. Using the log-transformed data C_*max*_, AUC_*0-t*_, AUC_*0-∞*_, AUMC_*tot*_, and MRT were calculated. The C_*max*_ of the test and brand was found to be 8.629±1.251μg/mL and 8.478±0.913μg/mL. The AUC_*0-t*_ and AUC_*0-∞*_ of the test and the reference were computed to be 20.890 ±2.2021μg/mL.h, 23.272 ±1.914 μg/mL.h and 19.850 ±2.911 μg/mL.h, 22.890 ± 2.110 μg/mL.h correspondingly. Two-way analysis of variance (ANOVA) test and two one-sided *t*-test (*p*>0.05; non-significant) were applied to assess the variation in the period, sequence, subjects, and treatment. Geometric mean ratios for above mentioned pharmacokinetic parameters of reference/test were found within the acceptable FDA limits of 80–125% using 90% CI. There was no inter and intrasubject variation (*p*> 0.05) that was observed. Therefore, the directly compressible aceclofenac (100 mg) test formulation and the commercial reference tablets were declared to be biosimilar.

## 1. Introduction

Bioequivalence studies are widely used for assessing the bio-similarity of pharmaceutical products. It is not only significant for the new product development (NPD) but also for the generic substitution and their interchangeability. The two formulations are said to be bioequivalent when insignificant differences are observed on the rate and extent of drug absorption (C_*max*_, AUC). According to the food and drug administration (FDA) recommendations, all generic and brands must meet the pharmaceutical standards in terms of identity, quality, purity, potency, and strength [1]. Considerable differences in prices have been extensively documented on comparative cost analysis of various brands and generics of pharmaceutical dosage forms [2, 3]. Significant monetary savings has been observed during the past decades in the US and UK by generic product substitution. About 77% and 83% of average savings in health care expenses had accounted respectively by US and UK governments with generic substitution practices [4, 5]. On the other side, it has been noticed that the quality attributes of the generic formulations are not meeting the standards as proposed by FDA, EMEA (European medicine agency), and other regulatory bodies. Moreover, various fake, adulterated, substandard, and spurious drugs were also marketed as an alternate generic option at affordable prices. Insufficient drug content and the poor quality of such products would definitely distrust the consultants and the patients as well for brand counterparts [6]. Pakistan being a lower-middle-income developing country, the healthcare system and facilities are not appropriately established [7]. Due to the lack of education and economic crises, peoples have no concern about quality rather the availability of medicines at a cheap cost. In this scenario, the in vitro and in vivo equivalence among innovators and generics must be established to overcome the therapy failures [8, 9].

Aceclofenac is a phenylacetic acid derivative, belongs to a class of non-steroidal anti-inflammatory drugs (NSAID). It is an antagonist of cyclooxygenase enzymes (COX-I and II) and widely prescribed for musculoskeletal disorders (osteoarthritis, rheumatoid arthritis, and spondylitis), body aches and dental pain [10, 11]. COX-II enzyme inhibitors are useful in different inflammatory complaints, but due to the cardiac toxicity, their utilization is limited. Beyond the fact, the reported safety and the tolerability of the aceclofenac are quite higher than other NSAIDs analogs. Henceforth, commonly recommended by the physicians of Asian and European countries [12]. Aceclofenac is a BCS (Biopharmaceutics Class System) class II drug with the log partition coefficient value of 2.170 [13]. The daily recommended dose of aceclofenac in an adult is 100 mg twice daily, necessitating adjustment of doses in hepatic impairments [10]. It is well absorbed as an unchanged form of oral administration in the stomach and upper intestinal region. The reported mean plasma drug concentration in human is ranged between 7–10μg/mL, achieved in 1–3 hours with mean elimination (T_1/2_) of approximately 4 hr (Rhim et al., 2008). It is metabolized by the cytochrome P450 enzyme system and excreted majorly in the urine.

The aceclofenac tablets are being commonly prescribed all over the world leading to increase in its demand. Availability of its cost-effective “substitutes” with standard quality attributes are becoming one of the prime concern nowadays. In this connection, aceclofenac formulation was prepared with minimum additives using simple technique of direct compression, making the overall product cost-effective. The present study aimed to characterize the newly designed directly compressible optimized aceclofenac IR formulation (F-15) in vivo. Moreover, to provide complete sets of information, the pharmacokinetic parameters are compared with marketed aceclofenac (100 mg) tablet brand. The in-vivo equivalence of both tablet formulations (test & reference) was evaluated after oral administration in healthy adult male subjects. As per FDA recommendation, bioequivalence testing of aceclofenac tablets was computed by Kinetica^®^ (version 5.1) software. Complete Latin square analysis of variance (ANOVA) was applied to assess the statistical differences between the products using a 95% confidence interval.

## 2. Material and methods

### 2.1. Materials

Aceclofenac (100mg) tablets manufactured by Novartis Pharma were purchased from the local market. All the necessary information including batch no., lot no., date of manufacturing and expiry was noted.

### 2.2. Chemicals and glassware

Aceclofenac pure was gifted by Sami Pharmaceuticals Pvt Ltd. Pakistan, methanol, and acetonitrile (HPLC Grade from Merck, Darmstadt, Germany), deionized water (obtained from Deionizer; Elga, High Wycombe, England) and ortho-phosphoric acid (Merck, Darmstadt, Germany) were procured. Borosilicate Glasswares (Pyrex, England) were used for analytical purposes.

### 2.3. Instruments

High performance liquid chromatography (HPLC, LC 20A, Shimadzu Corp., Kyoto, Japan), UV-spectrophotometric detector (SPD-20A, Shimadzu Corp., Tokyo, Japan), communication bus module (CBM 102, Shimadzu Corp.) connected with software (GC 20, Shimadzu Corp.), column coupled with a guard column (C-18 Nucleosil, Germany), vortex mixer (Whirl Mixer, England), pH meter (Jenway, England), centrifuge (Hereues, Germany), filtration assembly (Sartorious, Germany), microliter syringe (Hamilton, Switzerland), ultrasonic bath (E30H, Germany), membrane filters (Millipore, England) and Swinney filtration assembly (Millipore, England).

### 2.4. Methodology

#### 2.4.1. Formulation development of aceclofenac tablets by direct compression

Various formulation trials of aceclofenac (IR) tablets were prepared using different ratios of excipients, including avicel PH101, magnesium stearate, and croscarmellose sodium by direct compression technique. Design Expert^®^ software was utilized to generate their possible combinations (F1-F15 formulation runs) through the central composite rotation. The details of formulation compositions, physicochemical characterization, optimization, and stability studies are discussed in articles published previously [14, 15]. F15 formulation was selected for oral administration in healthy human individuals owing to the best tablet attributes and stability.

#### 2.4.2. Analytical method validation

*2*.*4*.*2*.*1*. *Chromatographic settings*. The content of aceclofenac in tablets was analyzed using the HPLC method. The mobile phase was a mixture of acetonitrile and deionized water (45:55) with a pH of 2.8 adjusted by 0.01M orthophosphoric acid solution. The flow rate was maintained at 1mL/min, and 20 μL of drug sample was injected and scanned at the wavelength of 276nm. The retention time for each sample was observed to be 10–11 minutes [16].

*2*.*4*.*2*.*2*. *Validation Parameters*. The previously reported HPLC method [16] was validated for the accuracy, selectivity, linearity, precision, stability, and sensitivity as per ICH guideline Q2A(R1)for method validation [17]. Plasma samples were prepared through protein precipitation by adding acetonitrile in equal proportion. The mixture was vortex for 2 min and centrifuged at 2000 rpm for 5 min. The clear supernatant was separated, and the filtered aliquot was injected in a 20 μL Rheodyne injector loop. Selectivity was assured by screening 6 blank plasma samples with LLOQ of aceclofenac. Linearity was established in a concentration ranged from 0.05μg/mL to 50 μg/mL. Three replicates of samples were used for calibration curve plotting. SD (standard deviation), % CV (coefficient of variance), and % accuracy were measured for each concentration. Intraday and inter-day precision were performed on three different drug strengths of 0.5, 5, and 10μg/mL. The selected drug solutions were assessed at different times on the same day and also for three successive days. Limit of detection (LOD) and lower limit of quantification (LLOQ) were estimated using four lower drug concentrations (0.015μg/mL, 0.025 μg/mL, 0.05μg/mL, 0.1μg/mL). The extraction efficiency of aceclofenac was determined through a comparative investigation of drug spikes in the plasma and mobile phase to establish absolute recovery. Three samples of different concentrations (0.5, 5 & 10μg/mL) were prepared in plasma and compared with those of mobile phase samples, and the results were expressed in % recoveries. The stability of the drug samples was estimated by “freeze and thaw cycle” in plasma using (0.5, 5 & 10μg/mL) strengths. Each time the samples were compared with the freshly prepared samples. Additionally, long term stability was performed by storing the samples at -40°C for three weeks. Samples were screened at the end of each week and compared to the respective concentration of fresh samples.

#### 2.4.3. Pharmacokinetic and bioequivalence study

*2*.*4*.*3*.*1*. *Study Design*. Bioequivalence study was conducted to measure the bio-similarity of prepared optimized aceclofenac tablet formulation (F15) with the marketed immediately release tablets according to the FDA guideline for bioequivalence studies of generic products [1]. The present study was a single-center with single-dose, randomized, two periods, two treatments, and cross over design conducted in a tertiary care hospital setting, with two weeks of washout period.

*2*.*4*.*3*.*2*. *Selection criteria for volunteers*. Blood sampling protocol and possible adverse reactions were explained to all the participants in detail in their local language (Urdu). A total of twelve male volunteers (healthy) were selected (coded as V1 to V12). Necessary physical measurements including weight, height, blood pressure, biomedical tests like complete blood picture, blood group testing, and medical history were documented prior to the study. Subjects were kept for one day in the tertiary care unit with all emergency facilities for the completion of the blood sampling.

*2*.*4*.*3*.*3*. *Ethics statement*. The study was approved by the Board of the Advanced Research (BASR) committee of the University of Karachi. The ethical approval was further granted from the Institutional Review Board Committee of Ziauddin University Hospital, Clifton, Karachi Pakistan (reference # 0760513 RBPHARM).

Participation of human voluneeers was entirely voluntary, and written signed informed consent was taken from each subject.

*2*.*4*.*3*.*4*. *Drug Administration*. Aceclofenac (100 mg) optimized formulation (F15) and the marketed (ACEMED) tablets were administered orally with a glass of water in fasted condition (>10hr) to subjects.

*2*.*4*.*3*.*5*. *Inclusion criteria and Exclusion Criteria*. Healthy male subjects with no previously reported drug- allergy, and normal findings of all physical, medical, laboratory assessment were included. Alcoholic, drug abusers, smokers, patients with a history of GIT bleeding or peptic ulcer, any hematological/ hepatic/ pulmonary/ renal/cardiovascular, and malignancies disorders were not recruited. Furthermore, females and participants taking any other medicine, or has been hospitalized or participated in any other bioequivalence study during the last three months were excluded from this study.

*2*.*4*.*3*.*6*. *Blood sampling protocol*. 5mL blood was withdrawn following an oral dose of directly compressed aceclofenac formulation and the commercial brand to each subject with overnight fasting (>10hrs). In both phases of investigation, the blood sample was taken before drug administration at 0 hr point and 0.5, 1, 1.5, 2, 2.5, 3, 3.5, 4, 6, 9, 12, 16, and 18 hrs. The second phase (two weeks washout period) of the study was conducted following the drug administration in a crossover pattern with a similar study protocol. The collected blood samples were centrifuged (at 3500 rpm for 10 minutes) to get plasma and then freeze at -20°C. The concentration of drug in plasma samples were examined by a validated HPLC method.

*2*.*4*.*3*.*7*. *Pharmacokinetic and statistical analysis*. Non-compartmental pharmacokinetic parameters were evaluated using software Kinetcia^®^ (version 5.1) (Thermoelectron Crop, U.S.A). The in- vivo data fitted to the oral two-compartmental model. Different non-compartmental parameters such as rate of absorption (K_*a*_), rate of elimination (K_*el*_), the maximum concentration of drug in plasma (C_*max*_), peak plasma concentration-time (T_*max*_), area under the plasma concentration curve (AUC), area under the moment curve (AUMC), clearance (Cl), elimination half-life (T_*1/2*_) and mean residence time (MRT) were measured for both test and reference products.

The bio-similarity of the test formulation against the reference brand was computed by comparing the C_*max*_, AUMC_*tot*_, AUC_*0-t*_, AUC_*0-∞*_ [18]. Schirmann’s two one-sided test of equivalence is documented in the past for estimating the difference between treatments. Moreover, the two-way ANOVA was applied to the Latin square study design to assess the inter and intrasubject variations during the treatment period.

*2*.*4*.*3*.*8*. *Drug tolerance*. In both phases of the study, drug safety or tolerability was noticed through adverse effects/reactions experienced by all the participants.

## 3. Results and discussion

It has been widely recognized that the generic products offer a better way to reduce the overall cost of the healthcare budget, especially in lower to middle economic countries. However, the quality, efficacy, and safety of these pharmaceuticals must be comparable in all aspects with the patent. In connection with this idea, various trials of aceclofenac immediate-release tablets had developed using central composite rotatable design. Different combinations of formulations using minimum and readily available additives were mixed and compressed directly to make cost effective, uncomplicated, less laborious, and reproducible techniques like of direct compression.

On the basis of acceptable physicochemical properties and stability, F-15 was selected to be the best formulation. The present study was designed to determine the significant pharmacokinetic parameters of the F15 formulation. Additionally, the bioequivalence of F15 formulation was also evaluated against the reference brand of aceclofenac 100mg tablets. The IRB ethically approved the pharmacokinetics and bioequivalence study protocols of Ziauddin university hospital. The clinical study was conducted in 12 Asian healthy male non- smoker individuals of an average age of 24.75 ± 7.60 years. The mean body weight and height of the subjects were 67.33 ± 3.12 Kg and 5.74 ±0.132 ft respectively. The mean blood pressure of the subjects were 83.166±5.03/ 120±4.85 mm Hg and 85.24 ± 4.23/ 118±5.56 mm Hg sequentially for phase I and phase II treatments. The entire study was performed in a tertiary care setting with 24 h emergency facility. Subjects were kept within the hospital till the completion of the two treatment courses.

**Fig 1:**
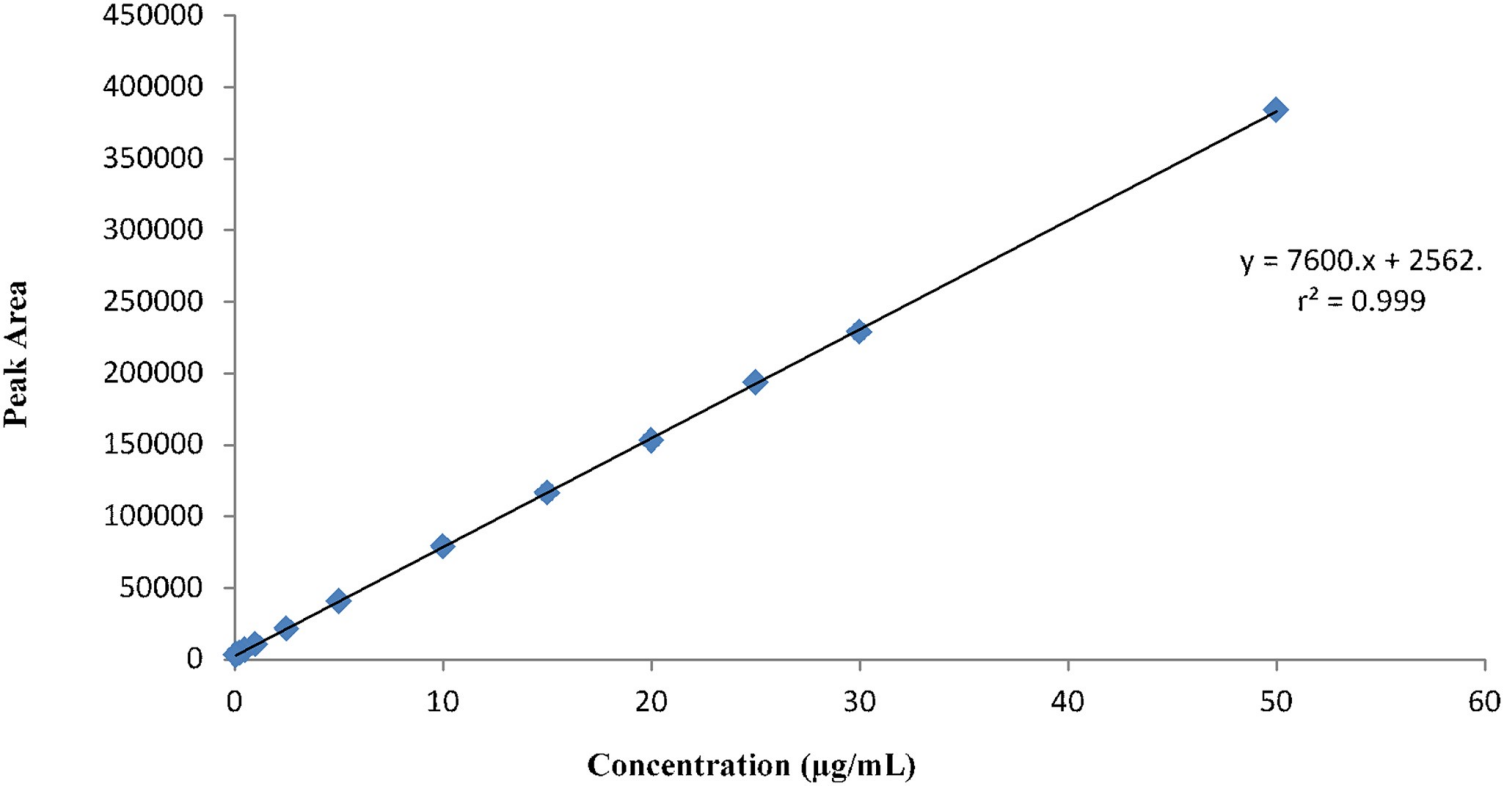
Mean plasma calibration curve.

A simple, bio-analytical HPLC method was validated as per FDA guidelines for the quantification of aceclofenac in plasma. The drug was extracted from the plasma samples prior to the analysis, with acetonitrile in a 1:1 ratio. This technique of protein precipitation is considered to be ideal due to the rapid and complete extraction of drug from the matrix. Therefore, in the development of bio-analytical procedures commonly used solvents such as heparin, orthophosphoric acid, trichloroacetic acid, and their combinations were used [19, 20]. The system suitability was observed at the middle-lower middle concentration of 5μg/mL by injecting 5 samples. The various system suitability parameters are given in Table 1. The linearity of the method was observed in three replicates by plotting the standard curve (10-points) of drug concentration and mean peak area. The method was found to be linear over a concentration ranged from 0.05μg/mL to 50μg/mL with a value of regression coefficient (*r*^2^) of 0.999 in the plasma (Fig 1). Intra and interday accuracy and precision were tested through analysis of three different concentrations of 0.5, and 10 mg/mL on three different consecutive days were 99.28%, 99.43% and 100.64% having % CV values of 1.665%, 1.11% and 2.194%, respectively (Table 2). LLOD is the lowest amount of drug that can be identified and taken when the signal to noise ratio is noticed thrice the baseline noise. LLOQ is considered to be the lowermost detectable and quantifiable strength against the standard. The lower limit of quantification (LLOQ) and lower limit of detection (LLOD) was calculated to be 0.05μg/mL and 0.025μg/mL sequentially. Freeze and thaw stability was assessed by spiking five thaw samples of three concentrations 0.5, 5, and 10μg/mL. The peaks were compared with the freshly prepared samples of the same strengths. All samples were found to be stable with %CV of 1.665, 1.635, and 0.26 correspondingly. The detail of the results is given in Table 3. Long term stability was determined by analyzing a fresh sample of the same concentration during their storage for two were sequentially found to be 98.40% (%CV1.036), 99.40% (%CV 0.49), and 99.53% (0.53%CV). The analytical recovery of the method was also evaluated at similar concentrations of stability for three days in three replicates. The % mean recovery was higher and calculated as 98.86%, 99.015% and 100.10% respectively for 0.5μg/mL, 5μg/mL and 10μg/mL. The validation results of the analytical method are similar to the studies reported in the literature for the assessment of various medicinal components in the biological samples [21–23].

**Table 1:** System suitability parameters.

Parameters	Mean(n = 5)	%RSD	Limit
**Retention Time**	12.36	0.640	<2
**Tailing Factor**	1.016	1.502	<2
**Area**	163254.7	0.342	-
**Theoretical Plate**	10297.33	0.078	-

**Table 2:** Inter and intra-day accuracy and precision of drug samples in plasma.

Concentration level (μg/mL)	Intra-day Assay (n = 5)			Inter-day Assay (n = 5)		
	Mean ± SD	Precision (% CV)	Accuracy (%)	Mean ± SD	Precision (% CV)	Accuracy (%)
0.5	0.49±0.007	1.306	99.80	0.496±0.008	1.665	99.28
5	5.05±0.047	0.924	101.12	4.97±0.055	1.11	99.43
10	10.06±0.044	4.338	101.46	10.064±0.220	2.194	100.640

**Table 3:** Stability and analytical recovery data of aceclofenac in plasma.

	Analytical recovery (3 days) (n = 3)			Freeze-thaw stability (3 cycles) (n = 5)			Long term stability (3 weeks) (n = 5)		
**Concentration (μg /ml)**	0.5	5	10	0.5	5	10	0.5	5	10
**Mean**	0.49±0.002	4.94±0.024	10.01±0.080	0.496±0.008	4.988±0.08	9.94±0.02	0.492±0.005	4.97±0.02	9.95±0.05
**Mean % recovery**	98.86	99.01	100.10	99.28	99.76	99.43	98.40	99.40	99.53
**% CV**	0.58	0.49	0.82	1.665	1.635	0.26	1.036	0.49	0.53

**Fig 2:**
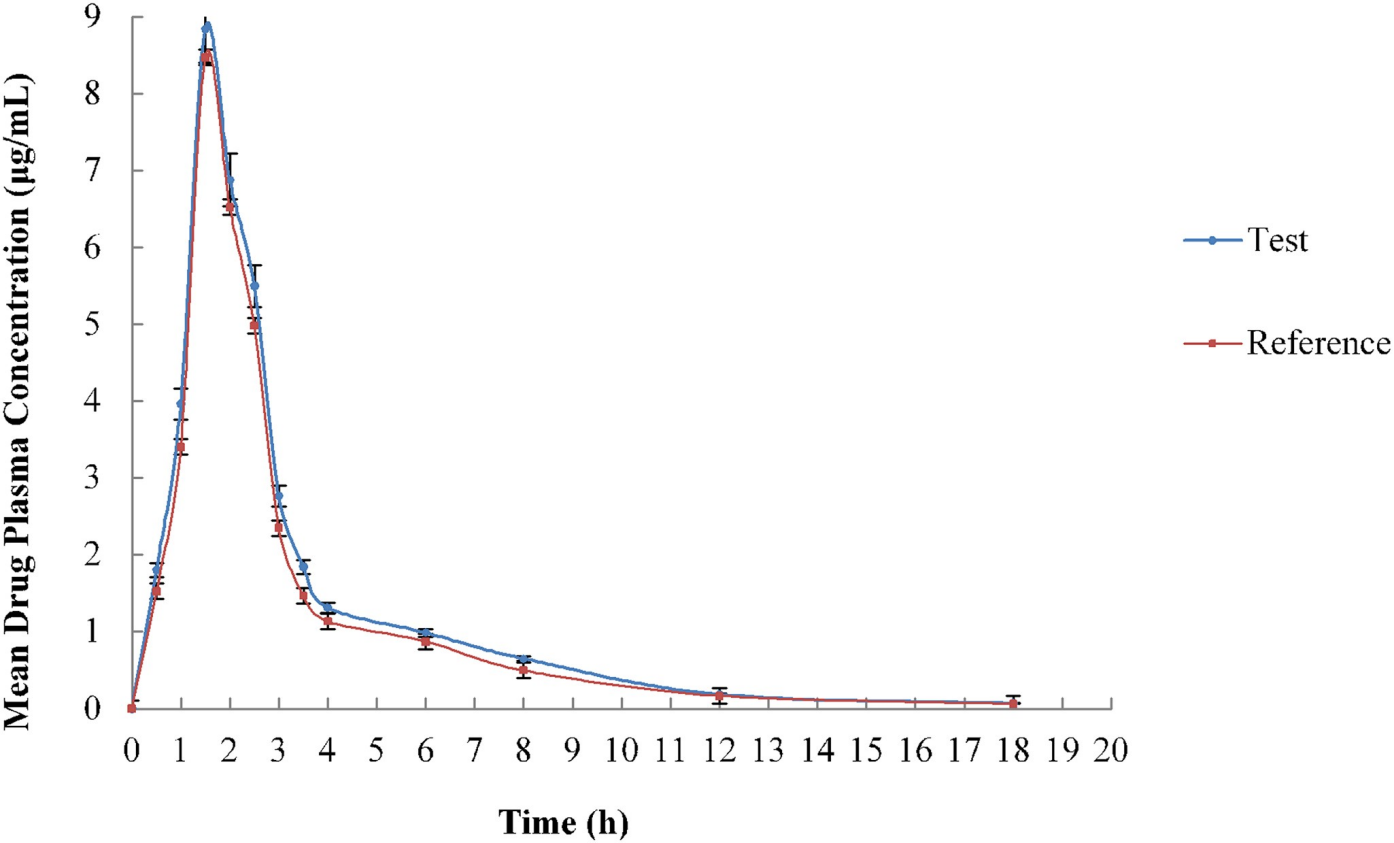
Mean plasma drug concentration of aceclofenac IR trial formulation and the reference product (n = 12).

The present study also estimates the significant PK parameters of the test formulation (newly developed) and the reference brand of aceclofenac (100mg) tablets. Randomized two-way cross over design with two treatments and sequences was adopted with a washout period of two weeks. The sequence of the phases and the treatments are given in Table 4. The Mean plasma drug concentration of both tablet formulations at various 12-time points is illustrated in Fig 2. Time vs. plasma concentration plots of the test and reference was observed to be overlapping due to insignificant differences of drug-in plasma. Plotted Area under the curve indicates the extent of drug absorption inside the body. The mean peak plasma concentration (see Table 5) computed for the developed formulation and the brand were in the close agreement with other previously reported researches dealing with the pharmacokinetic evaluation of aceclofenac (100 mg) formulations [16, 24, 25]. Ahmad et al. conducted a bioequivalence study for two aceclofenac tablet products tagged as A and B. The C_*max*_ of product A and B were 7.69 ± 0.36 μg/mL and 6.82 ± 0.13 μg/mL, observed at T_*max*_ of 3.0 ± 0.14 h and 2.94 ± 0.19 h correspondingly. The average value of drug clearance of product A and B were computed as 2.27 ± 0.10 Lh^-1^ and 2.18 ± 0.06 Lh^−1^, respectively [26]. Likewise, PK findings were also reported in Korean male volunteers dealing with the administration of the two commercial aceclofenac tablet generics.

**Table 4:** Sequence of two-way crossover design.

Group No.	Subject Codes	Treatment in Phase I	Wash out Period	Treatment in Phase II	Sequence
1	1,3,5,7,9,11	A*	Two-Weeks	B	AB
2	2,4,6,8,10,12	B**		A	BA

A*: Newly Developed Aceclofenac (100 mg) Tablet

B**: Marketed Brand of Aceclofenac (100 mg) Tablet

**Table 5:** Pharmacokinetic parameters of aceclofenac (100 mg) directly compressible trial formulation and marketed brand in healthy human subjects (n = 12).

Parameter	Directly compressible Aceclofenac Tablets Mean± SD	Marketed Aceclofenac Tablets Mean± SD
**K**_***a***_ ***(*h**^**-1**^**)**	2.068 ±0.166	2.079±0.245
**K**_***el***_**(h**^**-1**^**)**	1.017±0.079	0.994±0.081
**C**_***max***_**(μg/mL)**	8.629±1.251	8.478±0.913
**T**_***max***_**(hr)**	1.460±0.014	1.472±0.021
**AUC**_**0-t**_ **(μg/mL.h)**	20.890±2.201	19.850±2.911
**AUC**_**0-∞**_ **(μg/mL.h)**	23.272±1.914	22.89±2.116
**AUMC**_**tot**_**(μg/mL(h)**^**2**^**)**	74.1634±3.201	72.45±2.701
**CL (mL/h)**	4.98±1.048	4.63±2.084
**T**_***1/2***_ **(h)**	3.841±0.542	3.69±0.862
**MRT(h)**	3.615±1.125	3.4802±1.441

In comparison with the present results, higher values of C_*max*_ of the test (10.57 ± 3.07μg/mL) and sample (9.79 ± 2.94 μg/mL) at T_*max*_ of 1.92 h and 1.69 h were computed correspondingly [27]. The difference in results of similar pharmacokinetic studies conducted in the various geographical region may be associated with the CYP2C9 genotype (responsible for metabolism), which is variable in the different human populations [6]. The clearance of the drug from the body shows a correlation between drug in plasma and drug elimination rate from the body. The mean values for CL and T_*1/2*_ for newly developed F15 tablets and ACEMED tablets were determined to be 4.98±1.0484L/h; 3.841±0.542 h and4.63±2.084 L/h; 3.69±0.862h correspondingly. The values of rate constants (K_*a*_ and K_*el*_) for F15 and reference were found to be 2.068±0.166/h; 1.017±0.079/h and 2.079±0.245/h; 0.994±0.081/h respectively. The comparative pharmacokinetic measurements for the test and the reference formulations are presented in Table 5.

The bioequivalence among the preparations was evaluated, and the data were statistically analyzed through Latin Square (Kinetica^™^ User Manual, 2005). Products would have tagged to be biosimilar if the reference and test results fall between 0.80 and 1.25 using 90% CI after log transformation of data. The geometric mean ratios of AUC_*0-t*_. AUC_*0-∞*_, C_*max*_, AUMC_*0-tot*_, T_1/2_ and MRT of log-transformed data were computed as 1.003, 0.991, 0.992, 0.978, 0.977 and 0.957 respectively (Table 6). The effect of subject, formulation, sequence, and period was also evaluated. Two-way Latin square ANOVA was applied, and *P* values were found to be higher in all cases, reflecting a similar pattern of drug distribution (Table 6). Two-sided t-test with 90% confidence interval was applied on log-transformed data, and these values were found within the range of 0.8–1.25 (Table 6). The drug was well tolerated as none of the participants had reported any kind of pain, headache, vertigo, nausea, or discomfort in both treatment occasions. Therefore, aceclofenac (100 mg) tablets may be claimed as a safe drug of NSAID class.

**Table 6:** Log_n_ transformed pharmacokinetic parameters of aceclofenac test (F15) & reference for inter and intra subject variability (n = 12).

	C_*max*_ (μg/mL)	AUC_0-t_ (μg/mL.hr)	AUC_0-∞_ (μg/mL.hr)	AUMC_tot_ (μg/mL.(hr)^2^)	T_1/2_ (hr)	MRT (hr)
**Geometric Mean Ratio**	1.0033	0.991	0.992	0.978	0.9776	0.957
**Test Power**	0.8528	0.303	0.3192	0.361	0.1380	0.3285
CV*	0.0011	0.004	0.0041	0.006	0.1574	0.0949
RMSE**	0.0024	0.013	0.0127	0.029	0.0582	0.0638
***p*-value**						
**Period**	0.0883	0.699	0.7072	0.412	0.7363	0.0944
**Subject**	0.0757	0.384	0.3614	0.016	0.0570	0.0124
**Treatment**	0.0074	0.1418	0.1308	0.107	0.3652	0.1249
**Sequence**	0.3726	0.4811	0.4634	0.0027	0.1218	0.0036
***F*-Value**						
**Period**	3.5649	0.157	0.1494	0.730	0.1199	3.4135
**Subject**	2.5762	1.211	1.2589	4.194	2.8482	4.5584
**Treatment**	11.171	2.54	2.7089	3.128	0.8995	2.8044
**Sequence**	0.8714	0.535	0.5814	15.5373	2.8572	14.287

* Coefficient of Variation

** Root mean square Error

## 4. Conclusion

The validated bio-analytical HPLC technique with convenient extraction procedure was used for the quantification of aceclofenac in human subjects having regression (*r*^2^) value of 0.999. The pharmacokinetic parameters demonstrated the comparability in plasma drug exposure between the “test” and the “reference” formulations. These parameters were then used to assess the in-vivo equivalence in the products. F-15 directly compressible formulation was found to be bio-similar with the commercial brand of aceclofenac “ACEMED” of Novartis Pharmaceuticals. Geometric mean ratios of significant pharmacokinetic parameters were found to be within the acceptable limits of 0.8–1.25 (90%CI). No statistical variation (*p*> 0.05) was observed in subjects, sequence, treatment, and parameters. Both formulations showed good tolerance as no adverse reaction was reported by any male subject. Henceforth, the newly developed directly compressible formulation could be utilized clinically for the treatment of inflammatory and pain disorders, alternatively with cost-effectiveness.

## Supporting information

S1 Raw data (XLSX)
